# Shaping the aging brain: role of auditory input patterns in the emergence of auditory cortical impairments

**DOI:** 10.3389/fnsys.2013.00052

**Published:** 2013-09-17

**Authors:** Brishna Kamal, Constance Holman, Etienne de Villers-Sidani

**Affiliations:** Department of Neurology and Neurosurgery, Montreal Neurological InstituteMontreal, QC, Canada

**Keywords:** aging, auditory, noise, plasticity, inhibition, A1, parvalbumin, GABA

## Abstract

Age-related impairments in the primary auditory cortex (A1) include poor tuning selectivity, neural desynchronization, and degraded responses to low-probability sounds. These changes have been largely attributed to reduced inhibition in the aged brain, and are thought to contribute to substantial hearing impairment in both humans and animals. Since many of these changes can be partially reversed with auditory training, it has been speculated that they might not be purely degenerative, but might rather represent negative plastic adjustments to noisy or distorted auditory signals reaching the brain. To test this hypothesis, we examined the impact of exposing young adult rats to 8 weeks of low-grade broadband noise on several aspects of A1 function and structure. We then characterized the same A1 elements in aging rats for comparison. We found that the impact of noise exposure on A1 tuning selectivity, temporal processing of auditory signal and responses to oddball tones was almost indistinguishable from the effect of natural aging. Moreover, noise exposure resulted in a reduction in the population of parvalbumin inhibitory interneurons and cortical myelin as previously documented in the aged group. Most of these changes reversed after returning the rats to a quiet environment. These results support the hypothesis that age-related changes in A1 have a strong activity-dependent component and indicate that the presence or absence of clear auditory input patterns might be a key factor in sustaining adult A1 function.

## Introduction

Perceptual decline represents a universal component of the aging process across species, yet remains a poorly understood phenomenon. Perceptual deficits, particularly in the primary sensory cortices can commonly result in difficulties identifying fine details of stimuli, as well as a reduced ability to detect signals in noise (Divenyi and Haupt, 1997; Strouse et al., 1998). However, in recent years, much progress has been made in discovering the cellular and molecular correlates of slowed sensory processing. In aged rodents, for example, deficits in auditory processing have been linked to reduced inhibitory signaling (Seidman et al., 2002; Caspary et al., 2005), GABAergic transmission (Ling et al., 2005; Burianova et al., 2009), and parvalbumin positive (PV+) neurons (Ouda et al., 2008; Del Campo et al., 2012), and myelin (de Villers-Sidani et al., 2010; Tremblay et al., 2012). However, a recent upswing in research on cognitive training as therapy for age-related deficits has shown that many of these changes are reversible via specifically targeted training paradigms (de Villers-Sidani et al., 2010). The plastic nature of this inhibitory signaling, therefore, is unlikely to be the fundamental mechanism underlying age-related cognitive decline, and instead could represent a side effect of other complex neurological processes.

Hearing loss in younger animals has been extensively studied, and has led to many insights about the nature of such plasticity in the auditory system. Investigators have found strong evidence to suggest that peripheral damage leads to a down-regulation of inhibitory synapses, stemming from a decrease in statistically meaningful sensory inputs from the environment (Robertson and Irvine, 1989; Kotak et al., 2005; Takesian et al., 2009, 2013). This process, termed “negative plasticity,” serves to re-establish homeostasis in the auditory cortex, and may explain many of the features associated with altered processing accompanying damage to the auditory system (Syka, 2002; Takesian et al., 2009, 2011). Several authors have suggested that a similar mechanism may also contribute to age-related deficits in auditory processing, whereby altered input from the periphery (i.e., due to cochlear degeneration) leads to “noisy” information reaching the cortex (Mendelson and Ricketts, 2001; Caspary et al., 2005, 2008). Pioneering work from the Eggermont lab has shown that passive exposure to various forms of moderate-level noise is sufficient to cause dramatic changes in the A1 of adult cats, including disorganization of tonotopic indices and frequency tuning (Noreña et al., 2006; Pienkowski and Eggermont, 2010; Eggermont, 2013; Pienkowski et al., 2013). In rats, further work has shown that exposing young rats to continuous broadband noise could produce cortical patterns of broadened activation and temporal desynchronization in A1 similar to those seen in aged animals, yet with an absence of peripheral damage or degeneration (Zhou et al., 2011). Recently, similar exposure in adult rats has also been shown to distort tonotopic organization in A1 and compromise measures of pitch discrimination (Zheng, 2012). Thus, it appears that a simple change in the statistics of sensory inputs, essentially masking the majority of auditory patterns, can powerfully induce plasticity in the adult rat A1. Little is known, however, about the consequences of chronic noise on inhibitory circuits and processes that are specifically altered by aging. Further, although Pienkowski and Eggermont (2009) have shown that certain elements of A1 frequency representation can be remediated by placing adult cats in a quiet environment after noise exposure, much remains to be known about the mechanisms of inhibition involved in attenuation of noise-induced changes through such a recovery paradigm. If negative plasticity in response to altered input is indeed responsible for age-related A1 impairments, then to what degree may other plasticity contribute to recovery of normal auditory function in the aged or damaged auditory system?

To explore these questions, we exposed a group of young adult (6 months) rats to 8 weeks of low-grade broadband random noise to chronically mask auditory patterns reaching A1. The sound intensity level chosen for this exposure (65 dB SPL) is well below the threshold for potential hair cell damage. We hypothesized that after this exposure, the animals would demonstrate impaired auditory cortex tuning selectivity and temporal processing akin to deficits previously found in older rats (Caspary et al., 2008; de Villers-Sidani et al., 2010), accompanied by changes in inhibitory neuron populations and myelin. Finally, we posited that a return to a normal auditory environment would result in a spontaneous recovery of these noise-induced deficits, due to their plastic nature.

## Methods

All experimental procedures used in this study were approved by the Montreal Neurological Institute Animal Care Committee and follow the guidelines of the Canadian Council on Animal Care.

### Sound exposure

The young exposed rats were housed for 8 consecutive weeks in a sound attenuated chamber equipped with a speaker reproducing continuous (24 h/day, 7 day/week) broadband random noise covering 0.1–80 kHz and presented at 65 dB SPL via a calibrated speaker. The noise were generated with custom MatLab routines and played back via a MOTU UltraLite-mk3 Hybrid Interface sampling at 192 kHz.

### Mapping the auditory cortex

Fourteen female young (6 months old) and fourteen female aged (22 months old) Long-Evans rats were used for this study. For A1 mapping, the rats were pre-medicated with dexamethasone (0.2 mg/kg) to minimize brain edema. They were then anesthetized with ketamine/xylazine/acerpromazine (65/13/1.5 mg/kg, i.p.) followed by a continuous delivery of isoflurane 1% in oxygen delivered via endotracheal intubation and mechanical ventilation. Vital signs were continuously recorded using a MouseOx device (Starr Life Sciences, Holliston, Massachusetts). Body temperature was monitored with a rectal probe and maintained at 37°C with a hemothermic blanket system. The rats were placed in a custom designed head holder, holding the rat by the orbits, leaving the ears unobstructed. The cisterna magnum was drained of cerebrospinal fluid to further minimize cerebral edema. The right temporalis muscle was reflected, auditory cortex was exposed and the dura was resected. The cortex was maintained under a thin layer of silicone oil to prevent desiccation. Recording sites were marked on a digital image of the cortical surface.

Cortical responses were recorded with 32–64 channel tungsten microelectrode arrays (TDT, Alachua, Fl). The microelectrode array was lowered orthogonally into the cortex to a depth of 470–600 μm (layers 4/5), where vigorous stimulus-driven responses were obtained. The extracellular neural action potentials were amplified, filtered (0.3–5 kHz), sorted, and monitored on-line. Acoustic stimuli were generated using TDT System III (Tucker-Davis Technology, Alachua, FL) and delivered in a free field manner to right ear through a calibrated speaker (TDT). A software package (OpenEx; TDT, Alachua, FL) was used to generate acoustic stimuli, monitor cortical response properties on-line, and store data for off-line analysis. The evoked spikes of a single neuron or a small cluster of neurons were collected at each site.

Frequency-intensity receptive fields (RF) were reconstructed by presenting pure tones of 63 frequencies (1–48 kHz; 0.1 octave increments; 25 ms duration; 5 ms ramps) at eight sound intensities (0–70 dB SPL in 10 dB increments) to the contralateral ear at a rate of tone stimulus per second. Ten minute-long trains of tones pips with of 50 ms duration pips were presented at 5 pulses per second at a sound intensity of 70 dB SPL. Each train had a commonly occurring frequency (standard) with a probability of occurrence of 90% and a pseudo-randomly distributed oddball frequency pips presented 10% of the time with no repetition. The two frequencies in the train had constant separation of 1 octave and were chosen so they would be contained within the RF of the recorded neuron and elicit strong reliable spiking responses.

The stimulus used to estimate spectro-temporal receptive fields (STRFs) is based on the stimulus used in Blake and Merzenich (2002), and was created by adding independent tone pip trains at each 1/6th octave frequency bands between 0.75 and 48 kHz. Tone pips in each independent train were 50 ms long with 5 ms on and off ramps and occurred following a Poisson distribution with an average of 0.25 pip per second (average tone pip rate of 1 pip per 139 ms). The spectro-temporal density stimulus was presented continuously for 15 min.

### Immunohistochemistry

At the end of recording sessions, all rats received a high dose of pentobarbital (85 mg/kg i.p.) and perfused intracardially with saline followed by 4% paraformaldehyde in 0.1 M phosphate-buffered saline (PBS) at pH 7.2. Their brains were removed and placed in the same fixative overnight for further fixation and then transferred to a 30% sucrose solution, snap-frozen, and stored at −80°C until sectioning. Fixed material was cut in the coronal plane along the tonotopic axis of A1, on a freezing microtome at 40 μm. Tissue was incubated overnight at 4°C in either monoclonal or polyclonal antisera (For anti-PV: #P3088; dilution: 1:10,000; Chemicon International, Temecula, CA; for anti-MBP: ab62631; dilution 1:500; ABCAM). The following day, sections were washed and incubated in secondary anti-sera (for PV+, Cy2:#715-545-151; dilution 1:800; Jackson ImmunoResearch; for MBP, Alexa 647; dilution 1:800, Invitrogen). Tissue from young and aged rats were always processed together in pairs during immunostaining procedures to limit variables relate to antibody penetration, incubation time, and post-sectioning age/condition of tissue. A Zeiss LSM 510 Meta confocal microscope was used to assess fluorescence in the immunostained sections. Quantification of PV+ cells and MBP optical density (OD) was performed in Image J and MetaMorph imaging software (Molecular Devices Systems, Toronto, ON), respectively. To quantify Myelin OD, digital images of A1 cortical sections were taken with a 40× objective (Zeiss LSM 510). All quantification was assessed in 300–400 μm wide A1 sectors (rostral, middle, and caudal) per hemisphere extending from layer 1 to the underlying white matter. Data were then recorded as an averaged value for each case. The experimenters performing the histological measurements reported in this study were blind to the age of the animals.

### Electrophysiological data analysis

The characteristic frequency (CF) of a cortical site was defined as the frequency at the tip of the V-shaped tuning curve. For flat-peaked tuning curves, CF was defined as the midpoint of the plateau at threshold. For tuning curves with multiple peaks, CF was defined as the frequency at the most sensitive tip (i.e., with lowest threshold). Response bandwidths 10 dB above threshold of tuning curves (BW10) were measured for all sites. The CF, threshold, and BW10 were determined by using an automated routine developed in the MatLab environment (The MathWorks Inc., Natick, MA). A1 was identified based on its rostral-to-caudal tonotopy, reliable low-latency tone-evoked neuronal responses and relatively sharp V-shaped RF (Polley et al., 2006).

To generate A1 maps, Voronoi tessellation (a Matlab routine; The MathWorks) was performed to create tessellated polygons, with electrode penetration sites at their centers. Each polygon was assigned the characteristics (i.e., CF) of the corresponding penetration site. In this way, every point on the surface of the auditory cortex was linked to the characteristics experimentally derived from its closest sampled cortical site. The boundaries of the primary auditory cortex were functionally determined using published criteria (Bao et al., 2003). The normalized tonotopic axis of CF maps was calculated by rotating the map to make horizontal a linear function fit of the penetration coordinates using a least squares method. The tonotopic index (TI) was determined by computing the average minimum distance from each data point to the line connecting (0, 0) and (1, 1) after converting the logarithmic frequency range (1–48 kHz) to a linear range (0–1). We used the reverse correlation method to derive the spectrotemporal receptive field (STRF), which is the average spectrotemporal stimulus envelope immediately preceding a spike (STA) (Escabi and Schreiner, 2002). Only neurons with CFs well within the sound range of the stimulus were used. To enable comparisons between neurons, each STRF was normalized to the absolute value of peak activation of the STA. Total activation and inhibition strength was then calculated as the integral of the positive or negative area of the STA more than 2 standard deviations away from the baseline.

To compute the degree of neural synchronization in A1, we computed cross-correlation (CC) functions from each electrode pair by counting the number of spike coincidences for time lags of −100 to 100 ms with 1 ms bin size. These were then normalized by dividing each bin by the square root of the product of the number of discharges in both spike trains (Brosch and Schreiner, 1999). Neural events occurring within 10 ms of each other in two channels were considered synchronous. The degree of synchronization may be correlated with spike rates in a non-linear manner. For each pair of spike trains, we estimated the number of synchronized events if the two spike trains were not correlated, using *N*_A_N_B_Δ*T*, where *N*_A_ and *N*_B_ are the numbers of spikes in the two spike trains, Δ (=21 ms) is the bin size, and *T* is the duration of the recording (Eggermont, 1992; Bao et al., 2003). The strength of the synchrony was then assessed using a *Z*-score of the number of synchronous events: $Z=\frac{\text{number of syn events }-\;\frac{N_{A}N_{B}\Delta }{T}}{\sqrt{\frac{N_{A}N_{B}\Delta }{T}}}$. For neural synchrony recording, offline spike sorting using TDT OpenSorter (Tucker-Davis Technology, Alachua, FL) was performed to include only single units in the analysis.

Normalized responses to standard and oddball tones were obtained by dividing the average firing rate recorded in the 50 ms after the occurrence of each tone presentation by the average firing rate observed during the 50 ms after the first standard or oddball tone in the sequence. Asymptotes for standard and oddball responses were calculated by fitting exponential functions with a least squares method to the normalized response data from each recorded neuron. The method used to quantify probability coding in A1 has been previously described in detail Ulanovsky et al. (2003). Receiver operating characteristics (ROC) curves were calculated by plotting the true positive rate against the false positive rate of classification of oddball vs. standard the distribution of normalized firing rates as previously published (Britten et al., 1992; Dayan et al., 2001).

### Statistics

Statistical significance was assessed using unpaired two-tailed *t-tests* with Bonferroni correction for multiple comparisons. Data are presented as mean ± standard error to the mean (s.e.m).

## Results

### Comparing the effects of noise exposure and aging on frequency representation in A1

**Figure 1:**
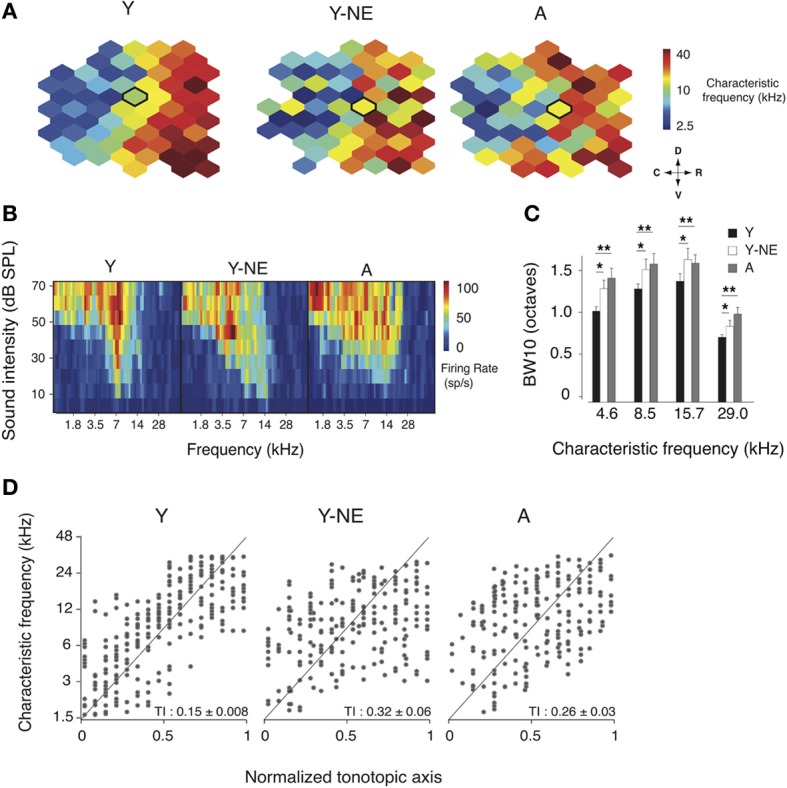
**Changes in frequency representation in the noise-exposed and aged A1. (A)** Representative A1 CF maps from the young (Y), young noise-exposed (Y-NE) and aged (A) experimental groups. **(B)** Representative cortical receptive fields (RFs) obtained for the neurons recorded in the center of the bolded polygons in the respective maps shown in A. **(C)** Average BW10 for all neurons recorded in each group and separated by CF. **(D)** CF of A1 neurons plotted against position on the normalized tonotopic axis of the corresponding recorded cortical site (all cortical sites pooled for each group). The average tonotopic index (TI) calculated for each individual A1 maps is shown (see Methods). Y: *n* = 6, 317 neurons; Y-NE: *n* = 5, 246 neurons; A: *n* = 6, 295 neurons. ^*^p < 0.05, ^**^p < 0.01: *t*-test.

In the adult rat A1, neurons' RF are usually sharp, V-shaped, and possess tuning that follows a smooth rostro-caudal gradient known as the tonotopic axis (Kelly and Sally, 1988; Zhang et al., 2001; Polley et al., 2006). Natural aging is associated with a broadening of A1 RFs and a disruption of this tonotopic axis (Turner et al., 2005; de Villers-Sidani et al., 2010). We investigated here whether an 8-week low-grade broadband noise exposure was sufficient to induce similar changes in A1 frequency representation. To do so, we examined the frequency tuning characteristics of A1 neurons in young adult (Y, *n* = 6), aged (A, *n* = 7) and young adult rats exposed to low-grade noise (Y-NE, *n* = 7). Representative examples of A1 characteristic frequency (CF) tuning maps in each group are shown in Figure 1. Noise exposure caused a significant RF broadening, as measured with BW10 (bandwidth 10 dB above threshold, see Methods) of neurons across the frequency spectrum (18% increase in BW10 compared to young naïve, *p* = 0.02–0.04, Figure 1C), with neurons tuned to low frequencies being slightly more affected. BW10 was also globally increased in the aged group across the frequency tuning range (38% increase on average compared to young naive, *p* = 0.002–0.01, Figure 1C). Low-frequency tuned neurons were also more affected in that group, with BW10 values similar to what has been previously reported in aged rats of a different strain (de Villers-Sidani et al., 2010). BW10 measures were not statistically different between the aged and noise-exposed groups (*p* > 0.2). The orderliness of frequency representation along A1's tonotopic axis was quantified using a tonopic index (TI) that assesses the degree of scatter in frequency tuning around an ideal logarithmic tonotopic progression (Zhang et al., 2001) (see Methods). Higher TI values imply more scatter. The TI was significantly elevated in both the aged and noise-exposed group compared to young controls (Y: 0.15 ± 0.008; Y-NE 0.32 ± 0.03, *p* = 0.003; A: 0.26 ± 0.03, *p* = 0.03, Figure 1D). An examination of the frequency distribution reveals that in Y-NE, the increase in TI is primarily due to the emergence of neurons with relatively low tuning (*CF* < 6 kHz) in more rostral sectors of A1. This effect was not present in aged rats, which displayed a more homogenous scatter in CF tuning. It should be noted that sound intensity thresholds in A1 were not significantly altered after noise exposure (*p* > 0.2). A few aged rats (<15% of those examined) showed significant increase in cortical thresholds attributable to peripheral hearing loss (usually in the high frequency >20 kHz range). These animals were excluded from this study.

### Spectro-temporal interactions in the noise exposed A1

**Figure 2:**
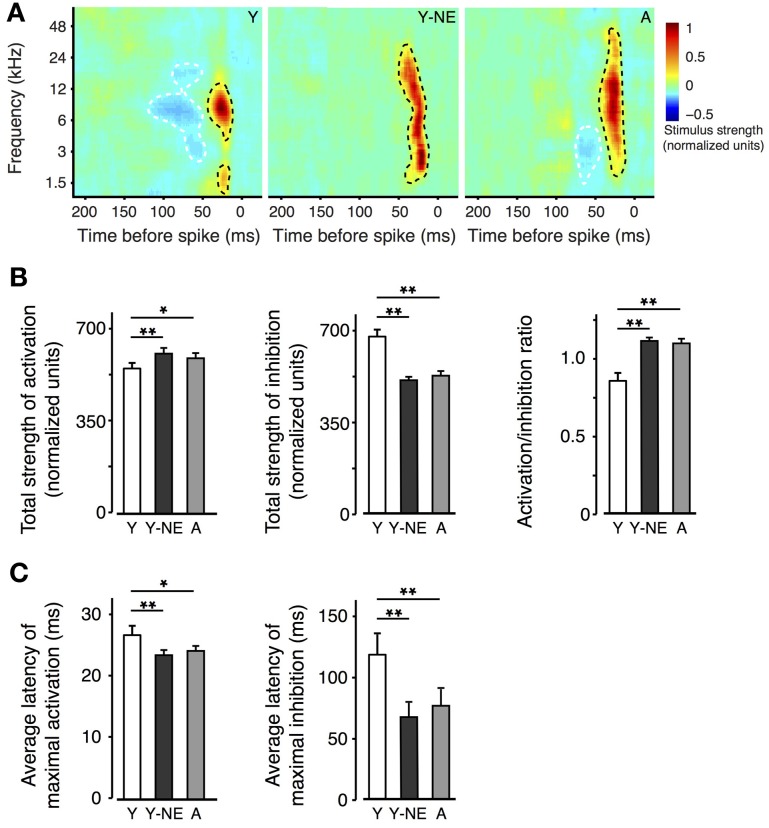
**Impact of noise exposure on spectro-temporal interactions. (A)** Representative spatio-temporal receptive fields (STRFs) obtained for single neurons in A1 in Y, Y-NE, and A groups. Positive (red, black dotted line) regions of the STRF indicate that stimulus energy at that frequency and time tended to increase the neuron's firing rate, and negative (blue, white dotted line) regions indicate where the stimulus envelope induced a decrease in firing rate. Note the smaller and shallower inhibitory areas in the aged noise-exposed and aged groups. **(B)** Total average strengths of activation, inhibition, and activation/inhibition ratio of the STRFs recorded. Note the increase in the activation/inhibition ratio in the Y-NE and A groups. **(C)** Average latency to maximal activation and inhibition in all experimental groups. (Y, number of STRFs recorded = 275; Y-NE = 208; A = 245). ^*^p < 0.05, ^**^p < 0.01: *t*-test.

The effect of natural aging on the sharpness of A1 neurons' tuning is thought to be partially due to reduced inhibitory influences from neighbouring A1 sectors. This phenomenon, also called “side-band inhibition,” can be quantified by reconstructing the spatio-temporal receptive fields (STRF) of A1 neurons using a dense broadband auditory stimulus (deCharms et al., 1998; Blake and Merzenich, 2002; Valentine and Eggermont, 2004; Noreña and Eggermont, 2005; de Villers-Sidani et al., 2010). The STRFs of neurons in each experimental group were computed using spike-triggered averaging (reverse correlation) of a “random chord” stimulus containing a spectrally and temporally dense sequence of random tone pips (see Methods). Representative STRFs obtained from each experimental group are presented (Figure 2A). Average inhibitory strength across each neuron population was computed, after the activation peaks of the STRFs were aligned, and response intensity was normalized according to the total strength of activation. Total STRF inhibitory area was, on average, 25% less in the naive aged group and young noise-exposed group, compared with the naive young group (Figure 2B; *p* = 0.0001–0.001). The ratio activation over inhibition was also computed for each neuron and found significantly elevated in the Y-NE and aged groups (*p* = 0.0002–0.001). This reduction in response inhibition in both groups was most apparent for stimulus frequencies less than 1 octave away from the neurons' best frequency and occurring 50–150 ms before neuron spiking. A clear broadening of the RF tuning was also observed and was reflected as an overall increase in area of activation in the STRF. This change, however, was relatively small compared to the change in inhibition (7 and 12% increase in NE and A, respectively, *p* = 0.02 and 0.03). A greater change in inhibition is also reflected in the activation to inhibition strength ratio, which was significantly increased in these two groups (Figure 2B). The latency to maximal activation and inhibition was also significantly reduced in the NE and A groups (average latency to maximal activation: Y: 27 ± 2 ms; Y-NE: 23 ± 2 ms, *p* = 0.002; A: 24 ± 2, *p* = 0.02; average latency to maximal inhibition: Y: 113 ± 18 ms; Y-NE: 68 ± 12 ms, *p* < 0.001; A: 74 ± 12 ms, *p* = 0.02 Figure 2C).

**Figure 3:**
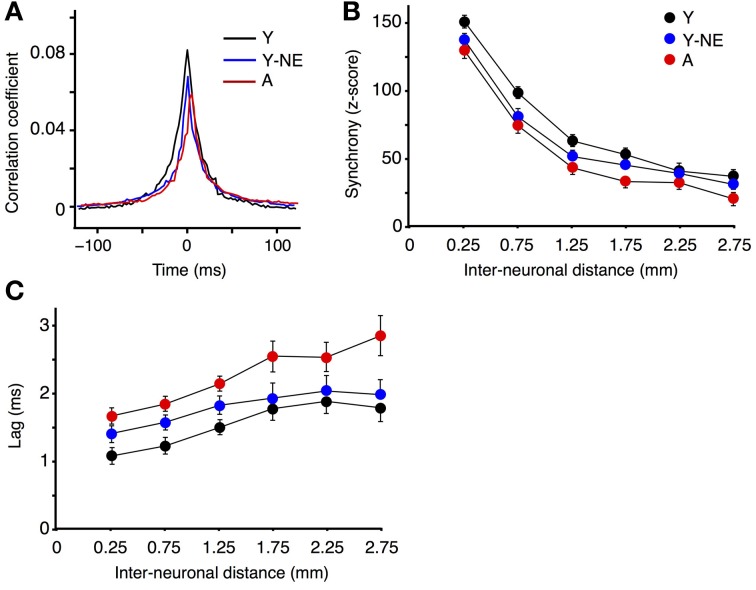
**Reduced A1 neural synchrony in noise-exposed and aged groups. (A)** Mean cross-correlation functions for all A1 neuron pairs with inter-neuronal distances less than 0.5 mm in Y, Y-NE, and A groups. **(B)** *Z*-score of neuronal firing synchrony (see Methods) as a function of distance for site pairs for all experimental groups. **(C)** Average time lag in absolute values of the peak of the cross-correlation function for all recorded pairs. (Y, number of site pairs: *n* = 513; Y-NE, *n* = 237; A, *n* = 263).

Corticocortical interactions in A1 were further studied by measuring CC functions and neural synchrony on the spontaneous discharge of individual A1 neurons at varying inter-electrode distances in all experimental groups. Higher CC and synchrony suggests stronger horizontal projections, indicative of more organized or efficient processing (Eggermont, 2007). The mean peak coefficient for all neuron pairs recorded at an inter-electrode distance of 0.5 mm or less was 14 and 27% lower in the noise-exposed and aged group, respectively, relative to the young controls (Y: 0.081 ± 0.008; Y-NE: 0.065 ± 0.01, *p* = 0.03; A: 0.058 ± 0.06, *p* = 0.006; Figure 3A). Furthermore, individual CC functions were on average 30% wider (width at half height of the peak) in the young control group compared to the noise-exposed and aged group (*p* < 0.001 in both cases). Neural synchrony measurements (see Methods) revealed that the impact of noise exposure was stronger on relatively short inter-neuronal distances (<1.25 mm), as it dissipated at distances of 1.75 or more (Figure 3B). In aged rats, however, the reduction in synchrony was significant at the longest inter-neuronal distances we could measure in A1 (2.75 mm). Noise exposure and aging both resulted in a slight but significant lag in the peak of the maximal correlation. This effect was seen for all interneuronal distances in the A group but only for neurons pairs separated by 1.25 mm or less in the NE group (average lag of CC function maximum: Y: 1.3 ± 0.1 ms; Y-NE: 1.6 ± 0.1 ms, *p* = 0.02; A: 1.9 ± 0.2, *p* = 0.01, Figure 3C).

### Impact of noise exposure on novel stimulus detection

**Figure 4:**
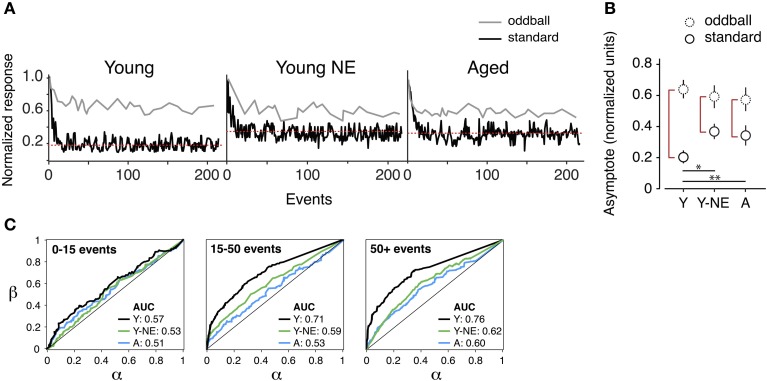
**Reduced deviant tone detection by A1 neurons in the noise-exposed group. (A)** Representative normalized responses of individual A1 neurons in the three experimental groups to standards (black line) and deviant tones or “oddballs” (gray line) as a function tone position in the stimulus sequence. The red dotted line represents the asymptote of the progressively suppressed response to standards. **(B)** Average asymptote computed for the response to oddballs and standards in all groups. Note the reduction in the difference between standards and oddball responses at steady state in the Y-NE and A groups (height of red vertical lines). **(C)** Average receiving operating characteristic curves computed from responses of individual neurons to oddball tone trains in all groups and for different time points in the tone sequence. AUC stands for “area under the curve.” Note that Y-NE and A groups do not reach the discrimination criterion (70%) (Y, number of neurons recorded = 275; Y-NE = 208; A = 245). ^*^p < 0.05, ^**^p < 0.01: *t*-test.

A1 neurons have the capacity to increase the salience of tones deviating in frequency from a sequence of monotonous stimuli (Ulanovsky et al., 2003). A reduction in the responses to these “oddball” tones is thought to be directly linked to the impairment in novel stimulus detection that follows natural aging (Ulanovsky et al., 2003; de Villers-Sidani et al., 2010). We compared the effect of noise exposure on deviant stimulus detection in A1 by presenting trains of identical, repetitive tones (standard or “distractors”) and introducing occasional deviant (oddball) frequencies. Exponential functions were fitted to the normalized response rates of A1 neurons to oddballs and standard tones in all experimental groups. This provided a quantitative measure of maximal suppression (asymptote, normalized units) of background tones and their separability from oddball tones (Figure 4). We found a significant reduction in the average suppression of responses to standard tones (mean standard asymptote of normalized response rate: Y, 0.20 ± 0.01; Y-NE, 0.38 ± 0.05, *p* < 0.001 relative to Y; A, 0.36 ± 0.07, *p* < 0.01 relative to Y) in both the noise exposed and aged groups. No significant difference in the overall magnitude of responses to oddballs was found in either group (*p* > 0.2). Overall, the effect of noise exposure and aging translated into a diminished response gap between standards and oddballs (asymptote *difference* between oddballs and standards; Y 0.43 ± 0.06; Y-NE 0.21 ± 0.08, *p* < 0.01 relative to Y; A; 0.22 ± 0.06, *p* < 0.01 relative to Y).

The difference in novel stimulus detection was quantified in the experimental groups by performing receiver operating characteristic ROC analyzes on response rates to oddballs and standards (see Methods, Figure 4C). This measure gives an indication of how an ideal observer would discriminate between the occurrences of an oddball or standard tone, solely based on the response magnitude of the recorded neuron. In young adult rats, the probability of a reliable discrimination (70% of correct classification, measured by the area under the ROC curve) was reached on average once 15 tones had been presented in the sequence (average successful classification rate probability: 0.71 ± 0.04), and was maintained thereafter. By contrast, for the aged and noise-exposed groups, this value remained significantly lower, under the detection criterion, even after the presentation of 200 tones in the oddball sequence (Y-NE: 0.60 ± 0.05, *p* = 0.04; A: 0.59 ± 0.07, *p* = 0.03).

### Reduction in PV+ cells and myelin in the A1 of noise exposed and aged rats

**Figure 5:**
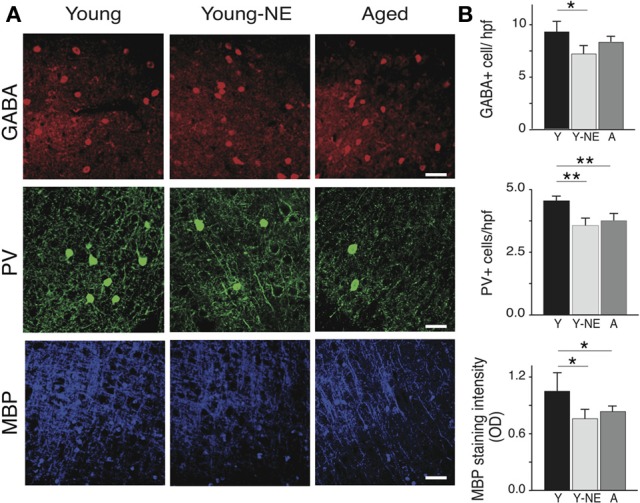
**Noise-induced changes in A1 PV+ interneurons and myelin.** Quantitative analysis of the average number in A1 of GABA and PV immunoreactive cells per high power field (hpf) and myelin basic protein (MBP) immunoreactivity in all experimental groups. **(A)** Representative high power photomicrographs of representative sections in all groups. **(B)** Average PV+ and GABA+ cell counts and average MBP staining fluorescence optical density in all groups (all layers averaged). Note the relative decrease in PV+ cell count and MBP staining in Y-NE and A groups. Number of hemispheres examined: Y = 8, Y-NE = 8, A = 10. ^*^p < 0.05, ^**^p < 0.01: *t*-test. Scale bar: 50 μm.

Parvalbumin positive (PV+) cortical neurons are part of a group of inhibitory inter-neurons that play an important role in stimulus selectivity and novel stimulus detection in sensory cortices (Beierlein et al., 2000; Fries et al., 2002; Lee et al., 2012), and aging is associated with a reduction in their numbers in the rat A1 (de Villers-Sidani et al., 2010). A progressive decline in brain myelin is also observed in the aging rat and human brain A1 (Itoyama et al., 1980; Steen et al., 1995; de Villers-Sidani et al., 2010), and is also thought to contribute to age-related cognitive decline (Peters, 2002). To determine if low-grade noise exposure can mimic the effect of aging on these structural elements of the cortex, we measured the density of PV+ and GABA+ cells and myelin basic protein (MBP) in A1 in our 3 experimental groups using standard immunofluorescence techniques (Figure 5A). Noise exposure resulted in a significant 30% reduction in PV+ cells in A1 (all layers pooled, *p* < 0.001), which was equivalent to the reduction observed in naive aged relative to young controls (25%, *p* < 0.01) (Figure 5B). The global count of inhibitory interneurons in A1 as evidenced by staining for GABA was also significantly lower in noise-exposed rats by 20% compared to young controls (*p* < 0.01, Figure 5B), but this was not the case for aged rat (Y: 8.73 ± 0.72 vs. 8.0 ± 0.58 GABA+/hpf, *p* = 0.2). Staining for MBP was also significantly reduced in A1 following noise exposure (44% relative to Y, *p* = 0.04), which was similar to what was observed in the aged group (38% *p* = 0.002 relative to Y).

### Experience-dependent reversibility of noise-induced and age-related A1 changes

**Figure 6:**
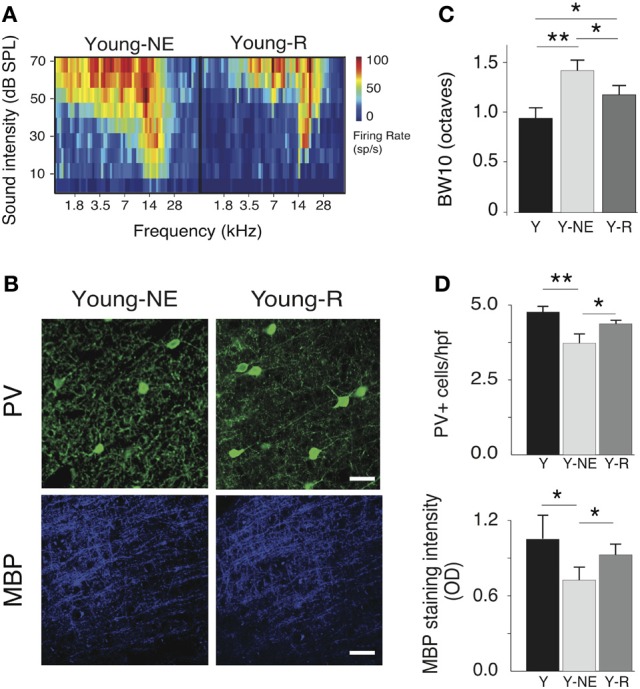
**Progressive recovery of A1 alterations after noise discontinuation. (A)** Representative RFs of A1 neurons in the Y-NE and young recovered (Y-R) groups. **(B)**, Average BW10 in Y-NE and Y-R groups (all recorded neurons pooled). **(C)**, Representative high power photomicrographs showing immunoreactivity to PV and MBP in Y-NE and Y-R. **(D)**, Quantitative analysis of the average number of PV+ cells and myelin density per high power field in all layers in the Y-R group, Y-NE and Y groups for comparison. Y: *n* = 6, neurons = 317; Y-NE: *n* = 5, neurons = 246, and Y-R: *n* = 4, neurons = 198. Number of hemispheres examined: Y = 8, Y-NE = 8, Y-R = 4. ^*^p < 0.05, ^**^p < 0.01. Scale bar: 50 μm.

To examine the reversibility of functional and structural noise-induced changes in A1, we placed a group of young adult rats (6 months old, *n* = 5) previously exposed to noise for 8 weeks in a quiet, noiseless auditory environment for an additional 8 weeks. We then mapped in this group's A1 responses to the same stimuli used in the noise exposed group, and quantified PV+ cell populations and myelin density. Average breadth of RFs in A1 was significantly less than after noise exposure but still slightly higher than in young controls (average BW10 Young-R vs. Y: 1.21 ± 0.11 vs. 0.95 ± 0.14, *p* = 0.05; Y-NE: 1.46 ± 0.11, *p* < 0.01, Figure 6B). A similar finding was obtained for the orderliness of the frequency representation gradient in A1, where the time spent in the noiseless environment did not appear to be sufficient for the tonotopic axis to return to normal (average TI Young-R vs. Y: 0.21 ± 0.02 vs. 0.15 ± 0.03, *p* = 0.03; Y-NE: 0.27 ± 0.03, *p* = 0.02, figure not shown). For all other functional measures examined, however, we found a complete normalization of A1 responses after discontinuation of noise exposure (figure not shown). These include STRF inhibitory and excitatory areas (*p* = 0.11 and *p* = 0.47, respectively), local neural synchrony (inter-neuronal distances < 0.5 mm, *p* = 0.32) and the asymptote of the response to recurrent tones in oddball sequences (*p* = 0.45). PV+ counts and myelin density also recovered partially in this group as shown in Figure 6.

## Discussion

Based on the results of our experiments, it appears that a prolonged masking of auditory input patterns with low-grade noise may be sufficient to cause numerous alterations in A1 normally associated with aging, such as tonotopic re-organization of A1 previously demonstrated by Zheng (2012) in a similar paradigm with younger animals. These changes, perhaps, represent the result of a form of negative plastic compensation akin to cortico-thalamic excitatory patterns accompanying induced hearing loss (Syka, 2002; Kotak et al., 2005), whereby inhibitory activity is reduced to compensate for “noisy” information from the environment. In older humans, it has been hypothesized that similar compensatory mechanisms may be linked to broadened patterns of cortical and subcortical activation observed by functional neuroimaging paradigms (Reuter-Lorenz and Cappell, 2008; Reuter-Lorenz and Park, 2010). However, these changes do not appear to be of a purely neurodegenerative nature in our experiment, as many aspects of normal auditory processing were recovered by young rats returned to a normal auditory environment.

Adult rats exposed to our broadband stimulus demonstrated significantly altered spectral and temporal processing profiles, as well as anatomical changes mirroring those previously found in aged animals (Mendelson and Ricketts, 2001; Caspary et al., 2008; de Villers-Sidani et al., 2010), and young adults exposed to continuous white noise (Zheng, 2012). To some extent, these patterns also mimic the diffuse excitatory nature of signaling of early critical periods (de Villers-Sidani et al., 2008; de Villers-Sidani and Merzenich, 2011). In fact, previous work using a very similar exposure paradigm in adult rats was shown to restore many characteristics of the critical period for noise exposure (Zhou et al., 2011), and extend it beyond its normal period of closure in young animals (Chang and Merzenich, 2003; Zhou et al., 2008; Zhou and Merzenich, 2009). In both cases, although the authors did not specifically test for abnormalities commonly associated with aging (i.e., response to oddball sequences), exposed animals demonstrated a BW10 profile very similar to that found in our results, pointing toward the capacity of unpatterned noise to provoke plasticity leading to widespread, non-specific excitation.

Despite several studies examining the impact of various forms of repetitive noise exposure on auditory processing, it is not yet clear by which mechanism this type of broadened excitation and diffuse responsiveness manifests itself. It appears that the phenomenon is primarily a cortical process (Ulanovsky et al., 2003), and thus, likely involves changes over several types of neuronal populations. For example, sustained excitation during recurrent sounds could stem from reduced suppression of recurrent excitatory input, perhaps from changes to the network of inhibitory interneurons in the cortex. Alternatively, the excitation could represent the failure of cells to reduce individual responses to incoming stimuli, as seen during stimulus-specific adaptation (Ulanovsky et al., 2003, 2004). Pienkowski and Eggermont (2012) have further suggested that such inhibitory changes could, in turn, impact inhibition on neighboring cortical fields, causing widespread changes in excitability across a major portion of A1. In either case, however, our STRF results are suggestive of large-scale plastic changes leading to deterioration of STRFs and weakening of strong post-activation suppression and side-band inhibition, previously found in aged rats (de Villers-Sidani et al., 2010). Similar mechanisms may also have played a role in our young rats' oddball response and BW10 profile, suggesting that during the course of exposure, large-scale, coordinated mechanisms of plasticity were mobilized. The reduction in post-activation suppression we observed after noise exposure is most likely due to a decrease in cortical inhibition (Eggermont, 1999; Wehr and Zador, 2005). Fast spiking interneurons (majority PV+) could be implicated more specifically in this deficit due to their influence on receptive field shape and strong gating of non-coincident inputs (Beierlein et al., 2000; Fries et al., 2002; Cardin et al., 2009; Lee et al., 2012) but the role of other inhibitory cells in in mediating this noise induced change can not be excluded. For example, somatostatin positive inhibitory interneurons have been shown modulate the gain of repetitive excitatory stimuli in hippocampal neurons (Kozhemyakin et al., 2013). Their exact role in A1 processing remains however, elusive.

Along with emergence of changes to spectrotemporal processing in A1, noise exposure also had an effect on the population of PV+ inhibitory interneurons and cortical myelin, which had both previously been shown to be reduced in the aged cortex (de Villers-Sidani et al., 2010). In the aged group, the total GABA+ cell count was not significantly decreased. Since all PV+ cell are also GABA immunoreactive, we interpret this as a reduction in PV expression with aging rather than cell death. An upregulation of PV+ expression in the aged A1 after auditory training also supports this idea (de Villers-Sidani et al., 2010). Noise exposure, however, led to a decrease both in GABA and PV expression, suggesting a role for reduced inhibitory neurotransmission in response to environmental noise. As with aging, noise also caused a reduction in MBP. The functional impact of reduced A1 MBP is unclear given the fact that we observed and overall reduction in tone-evoked latencies, which are also strongly influenced by inhibition (Calford and Semple, 1995). On the other hand, the increased lag in peak cortical synchronization we observed could in theory be secondary to a demyelination of cortico-cortical projections. The fact that PV and MBP expression normalized after the return to a quiet environment again indicates that this might occur here too through a down-regulation of protein production rather than cell actual cell death. Why GABA is reduced after noise exposure, yet not with aging is still unclear and the physiological consequences of reduced PV and MBP expression also remain to be clarified. However, in human populations, it is known that cortical inhibitory transmitters can change in complex ways throughout the lifespan (Sundman-Eriksson and Allard, 2006; Pinto et al., 2010), potentially contributing to changes in sensory processing in old age. Thus, they represent an exciting target for research on aging, and experiments are currently underway in our laboratory to explore this area.

Apart from examining the link between effects of low-grade noise exposure and age-related auditory deficits, this work has also provided some important insight on how plasticity in A1 may be mobilized to favor recovery from, and even reversal of such deficits. In humans, there is a growing literature centered around the use of training, or other enrichment strategies to mediate are-related auditory cognitive decline (Chislom et al., 2003; Alain et al., 2013; Anderson et al., 2013). However, many of the physical mechanisms of this recovery process remain to be elucidated, leaving room for experiments such as ours to determine what electrophysiological correlates are (and are not) subject to experience-dependent plasticity. Primarily, we have shown that noise-exposed young rats can regain A1 function more typical to their age group if returned to a more normal acoustic environment. However, it is still puzzling that despite improvements in BW10, oddball response and local firing synchrony, the TI of these animals failed to normalize. This result parallels the work of Pienkowski and Eggermont (2009), who had previously noted a similar disruption of tonotopic organization in the A1 of cats exposed to noise, despite 12 weeks in a quiet recovery environment. While the exact reasons for this discrepancy remain unknown it is possible that the tonotopic axis is most sensitive to non-patterned broadband noise, and requires a spectrally enriched, rather than quiet, recovery environment to return to normal. In any case, more work needs to be done to better characterize flexibility of the tuning curve across different exposure paradigms, particularly examining the time course of changes in the tonotopic map during recovery.

Questions additionally remain about the effect of enrichment on the auditory cortices of aged rats. Previously, several studies examining the effect of auditory enrichment on young animals have found it to improve many aspects of spectral and temporal processing (Engineer et al., 2004; Percaccio et al., 2005; Jakkamsetti et al., 2012). If increased environmental “noise” can, indeed, induce a form of negative plasticity as suggested by our results in noise-exposed young rats, then perhaps many age-related auditory processing deficits can be linked to a similar, yet more robust plastic response in the aging brain. This possibility certainly merits further study, and is currently being examined via several new ongoing projects.

Finally, further experiments are needed to define the precise nature of events precipitating impaired cortical processing in the aging brain. It is still unclear whether reduction in incoming regular sensory patterns [i.e., from peripheral degeneration such as the well-documented degeneration of cochlear hair cells (Crowley et al., 1972, 1973; Soucek et al., 1987; Seidman et al., 2002)] or “internal” noise caused by reduced inhibitory neurotransmitter cell populations in the cortex and/or brainstem leads to more significant decline. In the case of the latter scenario, it is additionally possible that decreased inhibition could set off a vicious cycle, at first compensating in an adaptive manner for altered input but subsequently resulting in the widespread desynchronization and slower temporal processing seen in our experiment. Though the development of an “aged” brain is doubtless a complex process, our experiments have provided a novel method to disentangle these factors via selective exposure/enrichment paradigms, and future work in our laboratory will aim to further explore the many facets of neurological change and adaptation in the aging brain.

In conclusion, environmental noise appears to have a powerful influence on creating auditory processing deficits akin to those in aged animals. This work supports the hypothesis that age-related auditory decline represents a form of negative plasticity, compensating for low-quality information arriving at the cortex (i.e., from damage to the cochlea or auditory pathways), and in the process, fostering diffuse and maladaptive patterns of excitation. Unfortunately, our experiment did not examine the behavioral correlates, but using a similar noise exposure procedure, recent work by Zheng (2012) has demonstrated that exposed rats had significantly impaired fine pitch discrimination, yet could adapt to perform behavioral tasks in noisy environments better than non-exposed controls. In addition, a small body of literature in human populations exposed to long-term broadband noise has hinted at the capacity of this type of exposure to be associated with a selection of cognitive impairments, ranging from difficulty with pitch discrimination to impairments in memory and attention (Gomes et al., 1999; Pawlaczyk-Luszczyñiska et al., 2005). Many other neuropsychiatric disorders are thought to result in a similar type of disorganized or “noisy” auditory processing, such as schizophrenia (Kim et al., 2009; Shin et al., 2012) or autism (Siegal and Blades, 2003; Kern et al., 2006), suggesting that similar mechanisms of plasticity may exist as in the aging brain. Fortunately, our work also suggests that these deficits are neither irreversible nor purely degenerative, which could have important implications for the remediation of brain processing impairments in these disorders. It is our hope that exposure-based paradigms such as ours may prove useful in modeling age or disease-related deficits in cortical processing, and that we may continue to gain a better understanding of changes in plasticity, and provide strategies of promote cognitive health across the lifespan.

## Author contributions

Brishna Kamal and Etienne de Villers-Sidani designed the experiments. Brishna Kamal performed the experiments. Etienne de Villers-Sidani and Brishna Kamal performed analysis. Constance Holman, Brishna Kamal, and Etienne de Villers-Sidani wrote the paper.

### Conflict of interest statement

The authors declare that the research was conducted in the absence of any commercial or financial relationships that could be construed as a potential conflict of interest.
